# Menstrual‐Associated Gastrointestinal Symptoms and Their Impact on IBS Diagnosis in Adolescents

**DOI:** 10.1111/nmo.70382

**Published:** 2026-06-22

**Authors:** Samantha Arrizabalo, Krisia Banegas, Carlos Alberto Velasco‐Benitez, Daniela Alejandra Velasco‐Suarez, Manuel Linares, Miguel Saps

**Affiliations:** ^1^ Division of Gastroenterology, Hepatology and Nutrition, Department of Pediatrics, Miller School of Medicine University of Miami Miami Florida USA; ^2^ Department of Pediatrics, Miller School of Medicine University of Miami Miami Florida USA; ^3^ Department of Pediatrics Universidad del Valle Cali Colombia

**Keywords:** adolescent, female, irritable bowel syndrome, menarche, Rome IV

## Abstract

**Objective:**

Irritable bowel syndrome (IBS) is the most frequent abdominal‐pain–predominant disorder of gut–brain interaction (DGBI). Because the pediatric Rome IV criteria require abdominal pain and stool changes for at least 4 days per month over 2 months, adolescents whose pain coincides with menstruation may be misclassified as IBS. We aimed to determine whether menstrual‐associated gastrointestinal symptoms contribute to the reported IBS prevalence.

**Methods:**

We conducted a cross‐sectional study of 3521 school‐aged females (10–16 years) from public and private schools in Colombia. Participants completed the validated Spanish Rome IV Pediatric Questionnaire for Gastrointestinal Symptoms, which included questions on temporal relation between pain, bowel habits, and menstruation. These symptoms were defined as occurring during days of active bleeding, without inclusion of a pre‐ or postmenstrual window.

**Results:**

Of 3336 analyzable surveys (mean age 13.3 ± 1.8 years), 2926 (87.7%) were postmenarcheal. Overall, 609/2926 (20.8%) met criteria for ≥ 1 DGBI, including 45 participants (1.5%) who met criteria for IBS. Among postmenarcheal participants with IBS, 62.2% reported abdominal pain and bowel‐habit changes exclusively during menstruation.

**Conclusions:**

A substantial proportion of adolescents meeting pediatric Rome IV criteria for IBS reported symptoms confined to menstruation, demonstrating a clinically relevant diagnostic overlap between dysmenorrhea and IBS. Because the Rome IV pediatric algorithm does not distinguish menstrual‐only pain from chronic abdominal pain, menstrual‐associated gastrointestinal symptoms may resemble those of IBS and complicate diagnostic decision‐making. Further studies are needed to clarify whether these presentations reflect menstrual‐related gastrointestinal symptoms alone or a distinct subset of DGBI.

## Key Points

- Irritable bowel syndrome (IBS) is a common condition that causes recurrent abdominal pain and changes in bowel habits such as diarrhea, constipation, or both.
- Dysmenorrhea and IBS can cause very similar gastrointestinal symptoms, and the current pediatric Rome IV criteria do not exclude symptoms that occur only during menstruation.
- In our study, 62% of postmenarcheal adolescents who met IBS criteria reported symptoms only during menses, emphasizing that symptom timing in relation to the menstrual cycle is critical for improving diagnostic accuracy.

## Graphical abstract

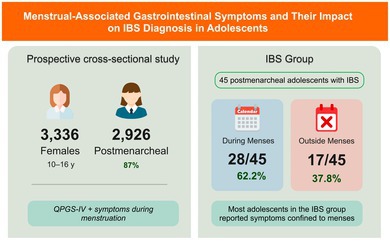

## 1 Introduction

Disorders of gut–brain interaction (DGBI) affect 25%–30% of children and adolescents worldwide [1, 2, 3]. These disorders can significantly impair daily functioning, school attendance, and social life, resulting in a considerable emotional and day‐to‐day burden on children and families [4, 5]. A meta‐analysis by Vermeijden et al. identified irritable bowel syndrome (IBS) as the most common functional abdominal pain disorder (FAPD) [3]. IBS is characterized by recurrent abdominal pain associated with changes in bowel movements and is diagnosed per the pediatric Rome IV criteria (Table 1) [6].

**TABLE 1:** Pediatric Rome IV diagnostic criteria for IBS [6].

Must include all the following:
Abdominal pain at least 4 days per month associated with one or more of the following: Related to defecation A change in frequency of stool A change in form (appearance) of stool
In children with constipation, the pain does not resolve with resolution of the constipation (children in whom the pain resolves have functional constipation, not irritable bowel syndrome)
After an appropriate evaluation, the symptoms cannot be fully explained by another medical condition
Criteria fulfilled for at least 2 months before diagnosis

Various risk factors have been implicated in the development and persistence of IBS and other DGBIs [7, 8, 9, 10]. Among them, sex has emerged as a risk factor, with studies in both adults and children reporting a higher incidence and persistence of IBS in females [11, 12]. This sex difference has been attributed not only to psychosocial and sensory factors, but also to the influence of sex hormones on gut motility, visceral sensitivity, and microbiota composition. Fluctuations in estrogen and progesterone can modulate bowel habits and pain perception, leading many females to experience abdominal symptoms during specific phases of the menstrual cycle [13, 14, 15]. In addition, prostaglandin release during menstruation can increase uterine contractility and influence intestinal motility and pain perception, contributing to gastrointestinal symptoms [16].

Dysmenorrhea, defined as painful menstruation, is common in adolescent females and often accompanied by abdominal pain and bowel changes [17, 18, 19]. Because these symptoms overlap with those used to diagnose IBS, diagnostic uncertainty may arise. The pediatric Rome IV criteria do not specify whether symptoms confined to menstruation should be excluded, raising the possibility that dysmenorrhea‐related gastrointestinal symptoms could fulfill IBS criteria [6]. Therefore, adolescents with abdominal pain and changes in bowel habits secondary to dysmenorrhea may meet IBS diagnostic criteria and represent a menstrual‐associated phenotype, potentially affecting IBS prevalence among adolescent females. Moreover, this diagnostic uncertainty may also result in inadequate and unnecessary treatments. We conducted a study to investigate this diagnostic overlap.

## 2 Aims and Hypothesis

### 2.1 Aim

Assess the relation of IBS‐like symptoms with menstruation among adolescent females.

### 2.2 Hypothesis

A subset of patients who meet the pediatric Rome IV criteria for IBS experiences gastrointestinal symptoms exclusively during their menstrual period.

## 3 Methods

### 3.1 Participants Recruitment

To ensure broad geographic and socioeconomic representation, this study was conducted across multiple regions of Colombia, encompassing both urban and rural school settings. Schools were selected to capture diverse ethnic and cultural populations from the Pacific, Andean, Amazon, and Caribbean areas. Most participating schools were public institutions, providing access to children from lower‐ and middle‐income backgrounds. All eligible students within participating schools were invited to participate in the study.

Eligible participants were female students aged 10–16 years. Participants were excluded if they had a known organic gastrointestinal, neurological, endocrine, or systemic disease that could explain abdominal symptoms, or if they had developmental or cognitive conditions that could interfere with comprehension of the questionnaire.

### 3.2 Consent and Assent

Information about the study's purpose and procedures was sent to the caretakers of all eligible students. Written informed consent was obtained from parents or legal guardians, and assent was obtained from each participant before enrollment. Participation was voluntary, and participants were informed that they could withdraw at any time without any consequences. No compensation or incentives were offered. The study adhered to national and international ethical standards for research involving minors.

### 3.3 Data Collection

Participants completed the Spanish version of the Questionnaire on Pediatric Gastrointestinal Symptoms—Rome IV (QPGS‐Rome IV) [20]. This validated tool assesses the frequency, duration, and characteristics of gastrointestinal symptoms to allow a positive, symptom‐based diagnosis of DGBI. The Spanish version used in this study was previously validated and employed in several epidemiological investigations by our research group [21, 22]. The translation and back‐translation process ensured linguistic and cultural accuracy. In addition to the core Rome IV items, the survey included questions on menarche and whether participants experienced abdominal pain and bowel changes exclusively during their menstrual period. Menstrual‐associated gastrointestinal symptoms were defined as those occurring during the days of active bleeding, with no additional pre‐ or postmenstrual time window or duration assessed. This classification was based on a single self‐report, without confirmation across cycles.

Before completing the questionnaire, participants received brief standardized instructions from trained staff to facilitate understanding. The survey was completed privately during school hours, with a research assistant available to clarify any questions. Completed questionnaires were collected and entered into a secure electronic database managed by the coordinating academic institution. Ten percent of the entries were randomly double‐checked for accuracy.

### 3.4 Statistical Analysis

Descriptive statistics were used to summarize demographic and clinical characteristics. Continuous variables are presented as means with standard deviations (SD), and categorical variables as frequencies and percentages. Differences between premenarchal and postmenarcheal participants were analyzed using 2 × 2 contingency tables, with Fisher's exact test applied when appropriate. Odds ratios (OR) with 95% confidence intervals (95% CI) were calculated. A two‐sided *p* value < 0.05 was considered statistically significant.

Prevalence rates for DGBIs and IBS were reported using two denominators: (1) the total number among postmenarchal participants who completed the questionnaire (*n* = 2926), providing population‐based estimates, and (2) the subset who fulfilled Rome IV diagnostic criteria for IBS to describe clinical characteristics. Statistical analyses were performed using *StataCorp LLC* (College Station, TX, USA; license 301606344270), with significance set at *p* < 0.05.

### 3.5 Ethical Approval

The study protocol was reviewed and approved by the institutional ethics committee in Colombia (approval #024‐2019). Data collection was conducted between September 2021 and August 2022. The research was performed in accordance with the Declaration of Helsinki and all applicable national and institutional regulations governing research involving human participants. This cross‐sectional study was reported in accordance with the STROBE guidelines.

## 4 Results

**FIGURE 1:**
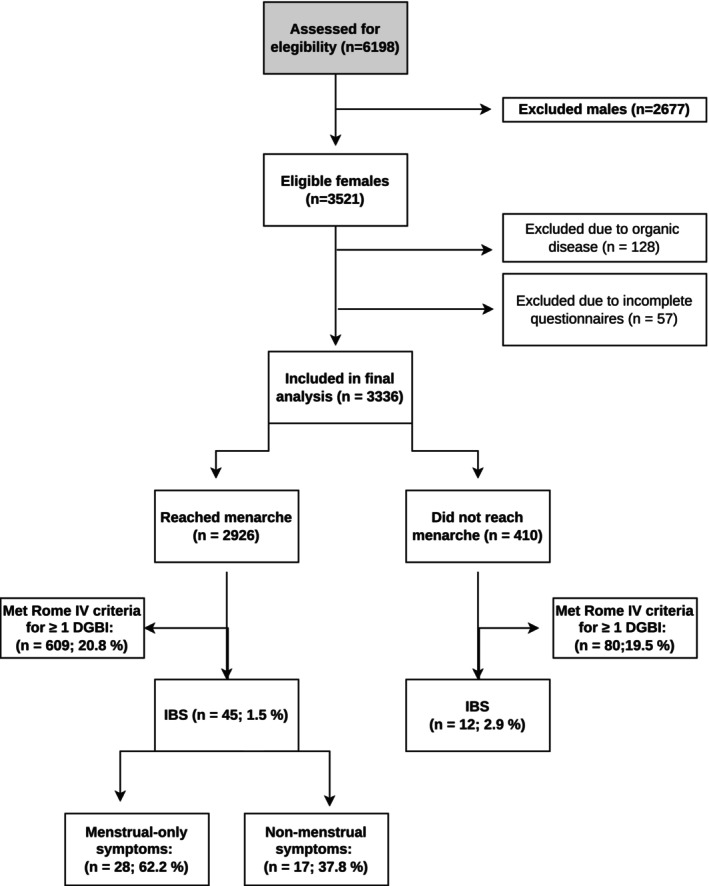
Flowchart of study enrollment and analysis. DGBI, disorder of gut–brain interaction; IBS: irritable bowel syndrome.

A total of 6198 students aged 10–16 years were screened across the participating schools. Of these, 2677 were males and excluded. Among the 3521 eligible females, 128 were excluded due to organic diagnoses and 57 due to incomplete questionnaires, resulting in 3336 fully completed surveys analyzed (Figure 1), corresponding to a participation rate of 91% among eligible students. The mean age of participants was 13.3 ± 1.8 years, and 66.3% were adolescents (13–16 years). Overall, 2926 (87.7%) had reached menarche, providing a large cohort in which to explore menstrual‐associated symptom patterns. Most participants attended public schools, and more than half self‐identified as of mixed racial background (Table 2).

**TABLE 2:** Demographics characteristics (*n* = 3336).

Characteristic	Overall (*n* = 3336)	Reached menarche (*n* = 2926)	Did not reach menarche (*n* = 410)
Age (years), mean ± SD	13.3 ± 1.8	13.6 ± 1.7	11.5 ± 1.0
Age group, *n* (%)			
School age (10–12 years)	1124 (33.7%)	780 (26.7%)	344 (83.9%)
Adolescents (13–16 years)	2212 (66.3%)	2146 (73.3%)	66 (16.1%)
Race/Ethnicity, *n* (%)			
Mixed	953 (54.5%)	820 (55%)	133 (51.8%)
White	515 (29.5%)	430 (28.8%)	85 (33.1%)
Black	209 (12.0%)	181 (12.1%)	28 (10.9%)
Indigenous	71 (4.1%)	60 (4%)	11 (4.3%)
School type, *n* (%)			
Public	2770 (88.0%)	2439 (83.4%)	331 (80.7%)
Private	566 (12.0%)	487 (16.6%)	79 (19.3%)
Region, *n* (%)			
Pacific	2843 (85.2%)	2476 (84.6%)	367 (90.5%)
Andean	270 (8.1%)	250 (8.6%)	27 (6.6%)
Amazonian	128 (3.8%)	116 (4%)	15 (3.7%)
Atlantic	95 (2.9%)	84 (3.0%)	7 (1.7%)
City of residence, *n* (%)			
Cali	1289 (38.6%)	1073 (36.7%)	216 (52.7%)
Tuluá	1213 (36.4%)	1116 (38.1%)	97 (23.7%)
Palmira	341 (10.2%)	287 (9.8%)	54 (13.2%)
La Unión	128 (3.8%)	113 (3.9%)	15 (3.7%)
Bucaramanga	154 (4.6%)	137 (4.7%)	17 (4.2%)
Florencia	126 (3.8%)	116 (4.0%)	10 (2.4%)
Corozal	85 (2.6%)	84 (2.9%)	1 (0.2%)

Among postmenarchal participants, 609/2926 participants (20.8%) met Rome IV criteria for at least one DGBI. Functional constipation was the most frequent diagnosis (16.7%), followed by IBS in 45 participants (1.5%).

Among the 45 postmenarcheal participants diagnosed with IBS, 28 (62.2%) reported abdominal pain and bowel‐habit changes exclusively during menstruation (OR 1.59, 95% CI: 0.83–3.12; *p* = 0.13). The remaining 17 (37.8%) reported pain occurring outside the menstrual period.

IBS subtypes differed according to symptom timing: among participants with menstrual‐associated gastrointestinal symptoms, the IBS‐unclassified subtype was most common (64.3%), whereas among those with IBS symptoms occurring outside menstruation, the mixed subtype was most common (41.2%) (Table 3).

**TABLE 3:** Distribution of IBS subtypes between premenarchal and postmenarchal females.

IBS Subtype	Premenarcheal (*n* = 12) *n* (%)	Postmenarcheal (nonmenstrual) (*n* = 17) *n* (%)	Postmenarcheal (menstrual‐associated GI symptoms) (*n* = 28) *n* (%)
IBS‐C (constipation‐predominant)	3 (25.0%)	4 (23.5%)	4 (14.3%)
IBS‐D (diarrhea‐predominant)	2 (16.7%)	3 (17.6%)	3 (10.7%)
IBS‐M (mixed)	4 (33.3%)	7 (41.2%)	3 (10.7%)
IBS‐U (unclassified)	3 (25.0%)	3 (17.6%)	18 (64.3%)

Abbreviations: IBS‐C, constipation‐predominant; IBS‐D, diarrhea‐predominant; IBS‐M, mixed; IBS‐U, unclassified.

## 5 Discussion

This study examined the overlap between dysmenorrhea and irritable bowel syndrome (IBS) in adolescents using the pediatric Rome IV criteria. We found that 62% of postmenarcheal adolescents who met IBS criteria reported abdominal pain and bowel‐habit changes exclusively during menstruation. This finding indicates that cyclic symptoms associated with menstruation can fulfill IBS diagnostic thresholds, creating diagnostic uncertainty for clinicians and suggesting meaningful diagnostic overlap between dysmenorrhea and IBS.

Menstrual‐associated gastrointestinal symptoms are common in adolescents, and several studies have reported increased gastrointestinal symptom burden among females with dysmenorrhea [23, 24]. Hormonal fluctuations across the menstrual cycle provide a plausible biological explanation for this pattern. Estrogen and progesterone receptors are widely distributed throughout the gastrointestinal tract, where they influence motility, visceral sensitivity, and pain signaling [13, 25, 26]. Estrogen can lower nociceptive thresholds, amplifying visceral pain responses, whereas progesterone may slow gastrointestinal transit and contribute to bloating or constipation. Prostaglandins released during menstruation increase uterine contractility but also enhance intestinal motility and visceral hypersensitivity, producing abdominal cramping and bowel‐habit changes that closely resemble IBS symptoms [26, 27, 28]. In parallel, emerging evidence shows that cyclical hormonal variations can alter gut microbiome composition, potentially contributing to symptom fluctuations throughout the menstrual cycle [25]. Together, these mechanisms support a possible hormonally mediated pattern of symptoms in which dysmenorrhea produces IBS‐like symptoms in the absence of chronic abdominal pain outside the menstrual period. Further studies are needed to determine whether these presentations reflect overlap with current pediatric DGBI criteria, a distinct menstrual‐related subtype, or a primarily gynecologic process accompanied by gastrointestinal symptoms.

Menstrual‐related symptom clustering is also observed in cyclic vomiting syndrome, in which a catamenial form has been described and responds to hormonal modulation [28, 29]. Such parallels provide further support for a model in which menstrual physiology can generate IBS‐like symptom patterns.

Differentiating dysmenorrhea‐related gastrointestinal complaints from functional abdominal pain disorders is clinically challenging. The pediatric Rome IV criteria require abdominal pain at least 4 days per month but do not specify whether symptoms confined to menstruation should be excluded from diagnosis [6]. In contrast, the adult Rome IV criteria mandate recurrent abdominal pain at least one day per week for 3 months [19], which typically excludes menstrual‐only presentations. This discrepancy highlights a structural limitation of the pediatric diagnostic algorithm when applied to menstruating adolescents. Our findings therefore raise the possibility that some adolescents diagnosed with IBS may instead reflect a dysmenorrhea‐IBS overlap rather than a chronic disorder of gut–brain interaction. Distinguishing between dysmenorrhea with gastrointestinal symptoms, a dysmenorrhea‐IBS overlap, and classical IBS is essential to avoid misdiagnosis, inappropriate labeling, unnecessary testing, and to guide focused therapeutic strategies.

This study has several strengths, including a large, community‐based sample drawn from diverse socioeconomic and geographic backgrounds and the use of a validated Spanish version of the Rome IV pediatric questionnaire administered under supervision. The study's focus on menstrual timing provides a clinically relevant perspective often overlooked in epidemiologic work on pediatric IBS. Nevertheless, limitations should be acknowledged. As a cross‐sectional study, it cannot determine whether menstrual‐only symptoms represent an early manifestation of IBS or a distinct hormonal phenotype. Although schools were selected to provide broad geographic and socioeconomic representation and participation was high, selection bias cannot be completely excluded. Symptoms were self‐reported and not supported by hormonal measurements, detailed menstrual cycle characterization, or data on contraceptive use, medication use, and psychosocial factors, all of which may have influenced reporting. The definition of menstrual symptoms was limited to days of active bleeding and based on a single assessment, without inclusion of a broader perimenstrual window, and may have been influenced by recall bias, limiting mechanistic interpretation. Additionally, the number of participants meeting IBS criteria was modest, limiting subgroup analyses and the precision of the estimates. Importantly, the observed association did not reach statistical significance (OR 1.59, 95% CI: 0.83–3.12; *p* = 0.13), and therefore these findings should be interpreted with caution. Despite these limitations, our findings identify a previously underrecognized diagnostic overlap between dysmenorrhea and IBS in adolescents.

In summary, the finding that most adolescents who met pediatric Rome IV criteria for IBS reported symptoms exclusively during menstruation could suggest a diagnostic overlap between dysmenorrhea and IBS. Clinicians should routinely assess the timing of abdominal pain relative to the menstrual cycle when evaluating adolescents with gastrointestinal complaints. These findings require confirmation in prospective studies using longitudinal symptom diaries across menstrual cycles. Such studies, together with hormonal profiling and microbiome analysis, may help determine whether menstrual‐only presentations reflect a hormonally mediated symptom pattern within DGBI or a diagnostic artifact of the pediatric Rome IV criteria.

## Author Contributions

Conceptualization: Carlos Alberto Velasco‐Benitez, Daniela Alejandra Velasco‐Suarez, Miguel Saps. Methodology: Carlos Alberto Velasco‐Benitez, Daniela Alejandra Velasco‐Suarez. Data curation/investigation: Carlos Alberto Velasco‐Benitez, Daniela A. Velasco‐Suarez. Formal analysis: Carlos Alberto Velasco‐Benitez, Daniela Alejandra Velasco‐Suarez. Writing – original draft: Samantha Arrizabalo, Manuel Linares, Krisia Banegas, Miguel Saps. Writing – review and editing: Samantha Arrizabalo, Manuel Linares, Miguel Saps. Supervision/project administration: Carlos Alberto Velasco‐Benitez, Daniela Alejandra Velasco‐Suarez. All authors approved the final manuscript and agreed to be accountable for all aspects of the work.

## Funding

The authors have nothing to report.

## Conflicts of Interest

The authors declare no conflicts of interest.

## Data Availability

The data that support the findings of this study are available from the corresponding author upon reasonable request.

## STROBE Checklist for Cross‐Sectional Studies

**Table:**

Item	Recommendation	Where addressed in manuscript
**Title/Abstract**		
1a	Indicate study design in title or abstract	Title, Abstract
1b	Provide informative and balanced summary of findings	Abstract
**Introduction**		
2	Background and rationale	Introduction (pg. 3)
3	State specific objectives	Introduction (last paragraph)
**Methods**		
4	Study design presented early	Methods (first paragraph)
5	Setting, locations, and dates	Methods—Participants
6a	Eligibility criteria and selection methods	Methods—Participants
7	Definitions of variables	Methods—Measures (Rome IV)
8	Data sources/measurement	Methods—Questionnaire description
9	Bias—efforts to address potential bias	Methods—Supervised administration
10	Study size—rationale	Methods—Sample frame
11	Handling of quantitative variables	Methods—Statistical analysis
12a	Statistical methods	Methods—Statistical analysis
12b	Subgroup/interactions	Not applicable (no subgroup testing)
12c	Missing data	Methods—Statistical analysis
12d	Sampling strategy	Not applicable (population‐based sample)
**Results**		
13	Participant flow/report numbers	Results—Figure 1
14a	Descriptive data	Results—Table 1
14b	Missing data	Results (not explicitly reported → note in limitations)
15	Outcome data	Results—Tables 2 and 3
16a	Estimates and precision (e.g., CI)	Results—Tables 2 and 3
16b	Confounders addressed	Discussion—diagnostic overlap
16c	Category boundaries	Clearly stated (Rome IV criteria)
16d	Sensitivity analyses	Not applicable
**Discussion**		
19	Key results summarized	Start of Discussion
20	Interpretation—caution about bias and limitations	Discussion
21	Generalizability	Discussion (external relevance emphasized)
**Other Information**		
22	Funding/role of sponsors	Title Page (Funding statement)
